# Data analysis in occupational health practice: lessons from experience

**DOI:** 10.1539/eohp.2026-0024

**Published:** 2026-08-20

**Authors:** You Hwi Song, Ken Kurisu

**Affiliations:** 1 OH Analytics, Inc., Tokyo, Japan; 2 Department of Cancer Survivorship and Digital Medicine, The Jikei University School of Medicine, Tokyo, Japan

**Keywords:** data analysis, machine learning, occupational health, problem-solving approach, sick leave, women’s health

Dear Editor,

One of the authors previously reported in this journal on a problem-solving approach to reduce sick leave due to mental disorders in a Japanese manufacturing company^1)^. Logistic regression was applied to the company’s data to identify factors associated with sick leave, and the results indicated several relevant factors, such as sleep disturbance. Subsequently, the authors analyzed data from another workplace using machine learning^2)^. The analysis showed that the most important predictor of sick leave due to mental disorders was the stress response score from the Brief Job Stress Questionnaire (BJSQ). Based on these results, we considered interventions in these workplaces to reduce sick leave^1,2)^. Building on these projects, similar analyses have been conducted in several companies. In this letter, we report lessons learned from these experiences.

In analyses of sick leave due to mental disorders, data on the BJSQ stress response score, which was the most important predictor in our previous project^2)^, were not always available due to company restrictions. In such cases, alternative measures, such as company-specific questionnaires on depressive and anxiety symptoms, were used. However, this often reduced the predictive accuracy of machine learning models, thereby leading to changes in intervention planning. In addition, anonymized data were sometimes created in collaboration with companies’ legal departments to comply with the Act on the Protection of Personal Information. Strict anonymization, such as categorizing age rather than using it as a continuous variable, substantially reduced predictive accuracy and complicated interpretation. Nevertheless, current stress symptoms were often identified as important predictors, as in our previous project^2)^, and were used to guide high-risk approaches.

Similar approaches have also been applied to data on women’s health and work productivity. Although attention to women’s health issues has been increasing, scientific evidence remains scarce^3)^, which may have increased demand for such analyses. Some analyses suggested that health literacy and self-care behaviors regarding menstrual and menopausal symptoms may be associated with productivity, informing proposals for workplace support to improve health literacy and self-care behaviors.

A recurring challenge was that companies often lacked a dedicated budget for such analyses or clearly defined rules for handling personal information for data analysis. Internal approval often took more than six months, and some projects could not be implemented. Conversely, projects were implemented relatively quickly when occupational health staff were highly motivated and had discretionary budget authority, or when appropriate procedures for handling personal information for data analysis were already in place. Data-use permission was obtained through discussions with corporate legal departments, under rules established by labor and management. Ethical considerations were addressed by limiting access to the analysis results and avoiding their use for discriminatory or other inappropriate purposes.

Therefore, projects similar to those we previously reported^1,2)^ can be applied in various companies in Japan. Unlike our previous projects conducted from within the company, these cases involved external support and extended beyond sick leave to broader occupational health issues. These projects suggest that data analysis helps to (1) clarify patterns in workplace health outcomes, such as sick leave and productivity, and their associated factors; and (2) develop potentially effective interventions based on analysis results. Nevertheless, because company-specific regulations on data use, available data, organizational resources, and workplace circumstances differ across companies, tailored approaches are required to ensure appropriate data handling, analysis, and intervention planning (Table 1). These lessons provide practical insights that can facilitate the application of data analytics to promote workers’ health. While data analyses are increasingly being reported elsewhere^4)^, occupational health professionals often face constraints on the time available for data analysis^5)^. Addressing this issue may help disseminate this approach more widely.

**Table 1.** Lessons learned from data analyses in multiple companies

• Company-specific regulations on data use should be confirmed, and data-use permission should be obtained in collaboration with the company’s legal department to ensure compliance with the Act on the Protection of Personal Information. • Lack of dedicated budgets or clear internal rules for the use of personal data can substantially delay approval, whereas motivated occupational health staff, discretionary budget authority, and established procedures for handling personal information for data analysis facilitate implementation. • Because available data differ across companies, the analysis design should be tailored to each company; the absence of key predictors can influence analysis results and change intervention planning. • Similar results were often observed across companies, such as current stress symptoms being important predictors of sick leave. • Strict anonymization, such as categorizing continuous variables, is sometimes required but can affect analysis results and complicate interpretation. • Data analysis can support company-specific intervention planning; however, each company’s resources and circumstances should be considered when planning implementation.
